# There is no association between a measure of clinical care and the response rate of GPs to postal surveys: A methodological study

**DOI:** 10.3109/13814788.2012.694861

**Published:** 2012-06-18

**Authors:** Sara Muller, Gwenllian Wynne-Jones, Rebecca Daniel, Samuel T. Creavin, Annette Bishop, Christian D. Mallen

**Affiliations:** 1 Arthritis Research UK Primary Care Centre, Keele University, UK; 2 North Bristol NHS Trust, Frenchay Hospital, Bristol, UK

**Keywords:** General practitioners, survey

## Abstract

**Background:** There has been much research into factors that can be modified to improve the response rates of general practitioners to surveys and to the demographic characteristics of those who do and do not respond. However, response is yet to be considered with respect to the quality of clinical care provided by GPs. In the UK, one measure of quality of care is the Quality and Outcomes Framework (QOF) score achieved by a general practice.

**Objective:** This study considers the association of QOF score with response to self-completion postal surveys of general practitioners.

**Methods:** Data are taken from two postal surveys of general practitioners (GPs) in the UK regarding their attitudes to osteoarthritis (OA) and sickness certification respectively. Logistic regression was used to assess the association between survey response and QOF score (as a proxy for quality of clinical care), adjusting for other characteristics of GPs and their practices (list size, number of partners, geographical region).

**Results:** There was no significant association of QOF score with survey response in either study, before or after adjustment for the other characteristics.

**Conclusion:** There is no evidence of an association between QOF score and the response of GPs to postal surveys. This gives reassurance that samples for studies of GP attitudes and practices should not suffer from response bias in relation to this core characteristic that represents the clinical achievement of their practice.

**Key Message**

• There was no association between a measure of the quality of clinical care provided by GPs (assessed using the Quality and Outcomes Framework score) and response of general practitioners to two postal surveys

## Introduction

Self-completion postal surveys are sent to clinicians in order to elicit their opinions of a variety of topics from attitudes to treatment modalities to ethics and new working practices (1). Achieving a good response to these surveys is the key to producing high quality research in these areas and to minimize the potential to introduce bias. A recent systematic review has shown published response rates to be variable, with an average of 61% (2).

There has been much attention given to modifiable factors that influence response rates to surveys of clinicians (e.g. 3 – 5), with randomized studies considering the use of personalized questionnaires, different font sizes and the use of incentives. Despite this growing body of literature, response rates in published surveys have remained relatively consistent over time (2).

It has previously been shown that several GP-related factors beyond the researchers’control are associated with whether or not a particular GP will respond to any given survey. These include GP age, whether they work in training practice, are a member of the Royal College of General Practitioners and the country in which they trained (6,7). A major influence on response rate, over which the researcher also has little control, is the substantive content of the surveys (8,9). However, there is currently no evidence as to whether GP response to surveys is associated with the quality of care provided by the practice. In the UK, the Quality and Outcome Framework (QOF) assesses the clinical performance of GPs and provides each practice with a score covering clinical and organisation factors in primary care, by which a proportion of GPs’pay is calculated (10). It was introduced in the UK in 2004 and can be seen as a proxy for the level of clinical care provided at the practice. The QOF contains four main components, known as domains. The four domains are: Clinical Domain; Organizational Domain; Patient Experience Domain; and Additional Services Domain. Each domain consists of a set of achievement measures, known as indicators, against which practices score points according to their level of achievement. Practices score points on the basis of achievement against each indicator, up to a maximum of 1000 points (10).

This paper utilizes data from two recent studies of general practitioners (GPs) in the UK in an attempt to answer the following question. What is the association between QOF score and response to a GP survey?

## Methods

### Sample

The data used in this article were taken from two separate studies conducted at the same research centre. The Prognostic Research III (PROG-RES III) Study was a postal survey of 2500 general practitioners (GPs) in the UK in 2010 to assess their attitudes towards discussing prognosis with patients with osteoarthritis in general practice (11). The overall response rate to this study was 31%, with 1% of those in the sample contacting the research centre to state that they did not wish to take part. The Sickness Certification in Primary Care (SCIP) Study was a postal survey of 2000 GPs, undertaken in 2008 to assess GP opinions of and attitudes towards sickness certification practices (12). The response rate in this study was 41% with 4% explicitly stating that they did not wish to take part. Both samples were randomly selected from the Binley’s database of all GPs working in the UK (13). In both studies, individual GPs were mailed a questionnaire and pre-paid return envelope to their practice address. In the PROG-RES III Study, non-responders were sent a reminder postcard after two weeks and additionally a reminder letter and repeat questionnaire after a further two weeks. In the SCIP Study, non-responders were sent a reminder letter and repeat questionnaire after two weeks only.

### Outcome of interest

For both studies, the outcome of interest was a response to the survey or not after the full sequence of reminders had been mailed. Those who contacted the research centre and declined to participate were considered non-responders, as were those from whom no response was obtained.

### Predictor of response

Quality Outcomes Framework (QOF) scores were available from the Binley’s database for all GPs in both study samples (10). Scores can range from 0 (lowest level of achievement) to 1050 (highest level of achievement), with practices receiving an average of £ 127.29 for every point achieved in 2010 - 2011, with the average score for a practice in England being 974 out of 1000 (10). QOF score was highly skewed (towards highest level of achievement) and so was divided into deciles for analysis. A higher decile indicated a higher QOF score and, therefore, a higher level of achievement.

### Potential confounders

Information was also available regarding the list size (number of registered patients) of each practice, and the number of partners in the practice. Number of partners in the practice was grouped as <1, 2, 3, 4, 5, >6. List size was normally distributed and so treated as a continuous variable for analyses. Practice postcode was used to allocate GPs to regions (14).

### Statistical analyses

Unadjusted logistic regression was used to assess the crude association of response to the surveys with QOF score. Multilevel logistic regression was used to model the association between QOF score and survey response adjusted for list size, number of partners in the practice and geographical region. This allowed for the possible clustering effect of region to be tested. In the presence of this effect, the multilevel model would be used for the final analysis. If this clustering effect was non-significant, a single-level logistic regression model would be used. List size was modelled as a continuous variable using a fractional polynomial to allow for a possible non-linear association with response. Likelihood ratio tests were used to assess the overall association of QOF score deciles with response in each model fitted. The PROG-RES III and SCIP Studies were considered separately, as it was not possible to ensure that the same GP had not been sampled for both studies. All analyses were conducted in Stata 12.0 (15).

### Ethical approval

Both the PROG-RES III and SCIP studies gained ethical approval from the North Staffordshire Local Research Ethics Committee.

## Results

Table I shows the associations of QOF score decile with response in the PROG-RES III dataset. There was a crude association between QOF score and response, with those with higher scores being more likely to respond (*P* = 0.0036), although this was not a monotonic association. On fitting a multilevel logistic regression model, there was no significant effect of clustering by region. A single-level logistic regression model adjusting for region, number of partners and list size attenuated the crude association (*P* = 0.2424).

**Table I:** Association of practice characteristics with survey response, *n* (%). QOF, Quality Outcomes Framework. Higher decile indicates higher level of achievement.

	PROG-RES (Overall response rate: 31%)		SCIP (Overall response rate: 41%)	
		
QOF score decile (range PROG-RES; Range SCIP)	Responders	Non-responders	Responders	Non-responders
1 (248–932; 462–934)	54 (22)	197 (78)	76 (41)	109 (59)
2 (933–964; 935–959)	78 (30)	179 (70)	79 (43)	105 (57)
3 (965–978; 960–973)	78 (30)	183 (70)	70 (38)	115 (62)
4 (979–985; 974–981)	73 (27)	201 (73)	71 (39)	113 (61)
5 (986–989; 982–987)	70 (33)	144 (67)	84 (45)	102 (55)
6 (990–992; 988–991)	77 (32)	166 (68)	92 (50)	91 (50)
7 (993–995; 992–994)	103 (35)	194 (65)	82 (44)	103 (56)
8 (996–997; 995–996)	75 (29)	182 (71)	71 (39)	113 (61)
9 (998–999; 997–998)	80 (38)	131 (62)	74 (40)	111 (60)
10 (1000–1000; 999–1000)	86 (37)	149 (63)	69 (38)	115 (63)
Number of partners				
≤^a^	144 (24)	449 (76)	90 (42)	123 (58)
2	114 (23)	375 (77)	93 (43)	124 (57)
3	121 (34)	238 (66)	109 (39)	168 (61)
4	117 (34)	224 (66)	122 (41)	173 (59)
5	102 (36)	185 (64)	129 (41)	188 (59)
6+	176 (41)	255 (59)	279 (41)	402 (59)
Practice list size (Mean SD)	7190 (4286)	6329 (3786)	8565 (4551)	8714 (4530)

Null hypothesis for all tests: no association with response.

PROG-RES, QOF χ^2^ = 23.99, *P =* 0.004; number of partners χ^2^ = 51.28, *P <* 0.001; region χ^2^ = 19.21, *P =* 0.014; list size *t =* —5.04, *P <* 0.001. Sickness certification, QOF χ^2^ = 11.28, *P =* 0.257; number of partners χ^2^ = 0.78, *P =* 0.978; region χ^2^ = 6.97, *P =* 0.540; list size *t =* 0.71, *P =* 0.476.

a One practice was recorded in the Binley’s database as having no partners.

In the SCIP Study, there was no crude statistically significant association between QOF score and survey response (*P* = 0.2609) (Table II). There was no clustering effect by region, and so a single-level logistic regression model was used to assess the adjusted association between QOF score and response. After adjustment, there remained no significant association, with confidence intervals for each decile of QOF score overlapping.

**Table II:** Adjusted association of practice characteristics with survey response: Results from a multivariable logistic regression model. QOF, Quality Outcomes Framework. Higher decile indicates higher level of achievement. Odds ratio for practice list sizes refers to increase in odds for every 1000 patients on the list.

	Odds ratio (95% CI)	
	
QOF score decile	PROG-RES	SCIP
1	1	1
2	1.45 (0.96, 2.18)	1.07 (0.70, 1.64)
3	1.29 (0.86, 1.95)	0.89 (0.58, 1.37)
4	1.06 (0.70, 1.61)	0.91 (0.59, 1.39)
5	1.42 (0.93, 2.18)	1.18 (0.77, 1.82)
6	1.28 (0.84, 1.95)	1.47 (0.96, 2.25)
7	1.42 (0.95, 2.13)	1.16 (0.75, 1.75)
8	1.09 (0.72, 1.66)	0.89 (0.57, 1.34)
9	1.52 (0.98, 2.34)	0.96 (0.62, 1.47)
10	1.47 (0.97, 2.25)	0.87 (0.56, 1.34)
Number of partners		
≤1	1	1
2	0.90 (0.67, 1.20)	1.01 (0.67, 1.51)
3	1.47 (1.07, 2.02)	0.80 (0.53, 1.19)
4	1.48 (1.04, 2.10)	0.99 (0.65, 1.50)
5	1.57 (1.05, 2.34)	0.95 (0.62, 1.46)
6+	1.96 (1.25, 3.06)	1.05 (0.66, 1.67)
Practice list size	1.00 (0.96, 1.04)	0.99 (0.95, 1.02)

CI, confidence interval; SD, standard deviation.

^a^One practice was recorded in the Binley’s database as having no partners.

## Discussion

There is no evidence of an association between GP response to a postal survey and QOF score. Any apparent association in the PROG-RES III dataset was attenuated by adjustment for other practice characteristics. This is likely to be reassuring to researchers who conduct studies of GPs, as it suggests that there will be minimal levels of response bias in terms of the clinical performance of the GPs in their sample who do and do not respond. In studies of attitudes and practices, this is likely to be of key importance.

This paper has drawn on two studies to examine the same research question: is practice QOF score associated with the responses of GPs to a survey? The paper found there to be no association. The fact that the find-ing from the PROG-RES study is broadly replicated in the SCIP study, suggests that the lack of association between QOF score and response rate may be generalizable. The surveys dealt with different subject matter: attitudes towards discussing prognosis with patients with osteoar-thritis and sickness certification provision in general practice. This has previously been shown to be a key factor in influencing the response rate to a survey (7). The questionnaires were also of differing lengths (PROGRES III Study: eight pages of questions, SCIP Study: five pages of questions), and it has been shown that a response is more likely with shorter surveys (1).

A potential limitation of this study is that it is possible that some GPs in the studies worked in the same practices, thereby making them more similar than would be expected by the statistical analyses. This could have been avoided through the use of a multilevel model where GPs were grouped within practices. However, the small number of GPs to whom this could have applied would make this method of analysis problematic and it is unlikely that any correlation between GPs has significantly affected the results obtained, as one would have to assume that practice-level characteristics heavily out-weight individual characteristics in influencing the decision to respond.

One might question the validity of QOF score as a measure of quality of care. However, this has been deemed to be such a measure by the UK government, and as such it is used to assess practice performance. Furthermore, this is the only proxy measure of clinical care provided by general practices currently available in a standardized way across the country.

A further consideration in the association between non-modifiable GP characteristics and response to surveys might be the socio-demographic make-up of the practice, but this information was not available for the PROG-RES III and SCIP Studies. A further limitation of this study is the inability to account for other factors that are known to contribute to the decision of a GP to respond (age, country of training, time since qualification) and on which data were not available from the Binley’s database (13). However, it seems unlikely that these factors would truly confound the association between QOF score and response, especially after adjustment for practice list size and number of partners, which give an indication of overall burden on the GP.

## Conclusion

The lack of association of a response to survey of GP attitudes with a measure of the quality of clinical care is reassuring. Further investigation of this finding in surveys carried out by other research centres and covering different substantive clinical areas would provide further reassurance.

## Funding

This work was supported by Arthritis Research UK (PROGRES study, CDM – grant number MO669), The North Staffordshire Medical Institute (SCIP Study), National Institute for Health Research (GW-J, RD), National School for Primary Care Research (SM).
